# Supplementary material for: Long-term fluorescence live imaging of honeybee embryos using light sheet fluorescence microscopy and halocarbon-based liquids
Source: Biol Open. 2025 Sep 26;14(9):bio062151. doi: 10.1242/bio.062151 (PMC12505272; doi:10.1242/bio.062151)
Supplement: Supplementary information [file biolopen-14-062151-s1.pdf]

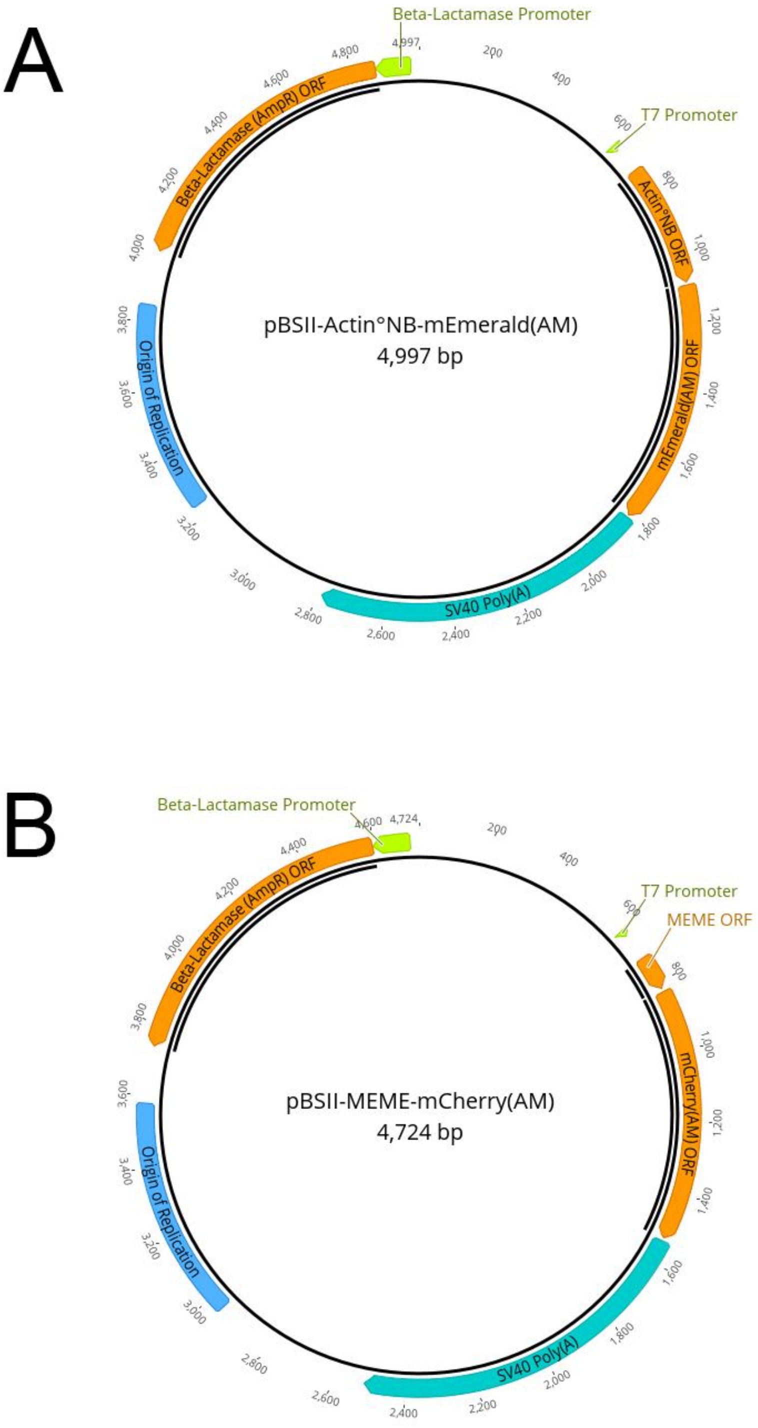

Fig. S1. *In-vitro* mRNA synthesis plasmids used in this study. (A) Plasmid map of pBSII-Actin°NB-mEmerald(AM). The T7 promoter-derived mRNA encodes mEmerald-labeled anti-actin nanobodies to visualize filamentous actin. **(B)** Plasmid map of pBSII-MEME-mCherry(AM) as an example for the nine open-access plasmids. The T7 promoter-derived mRNA encodes a mCherry-labeled GAP43 membrane anchor tag with an extended linker sequence to visualize the cell membranes.

Biology Open • Supplementary information

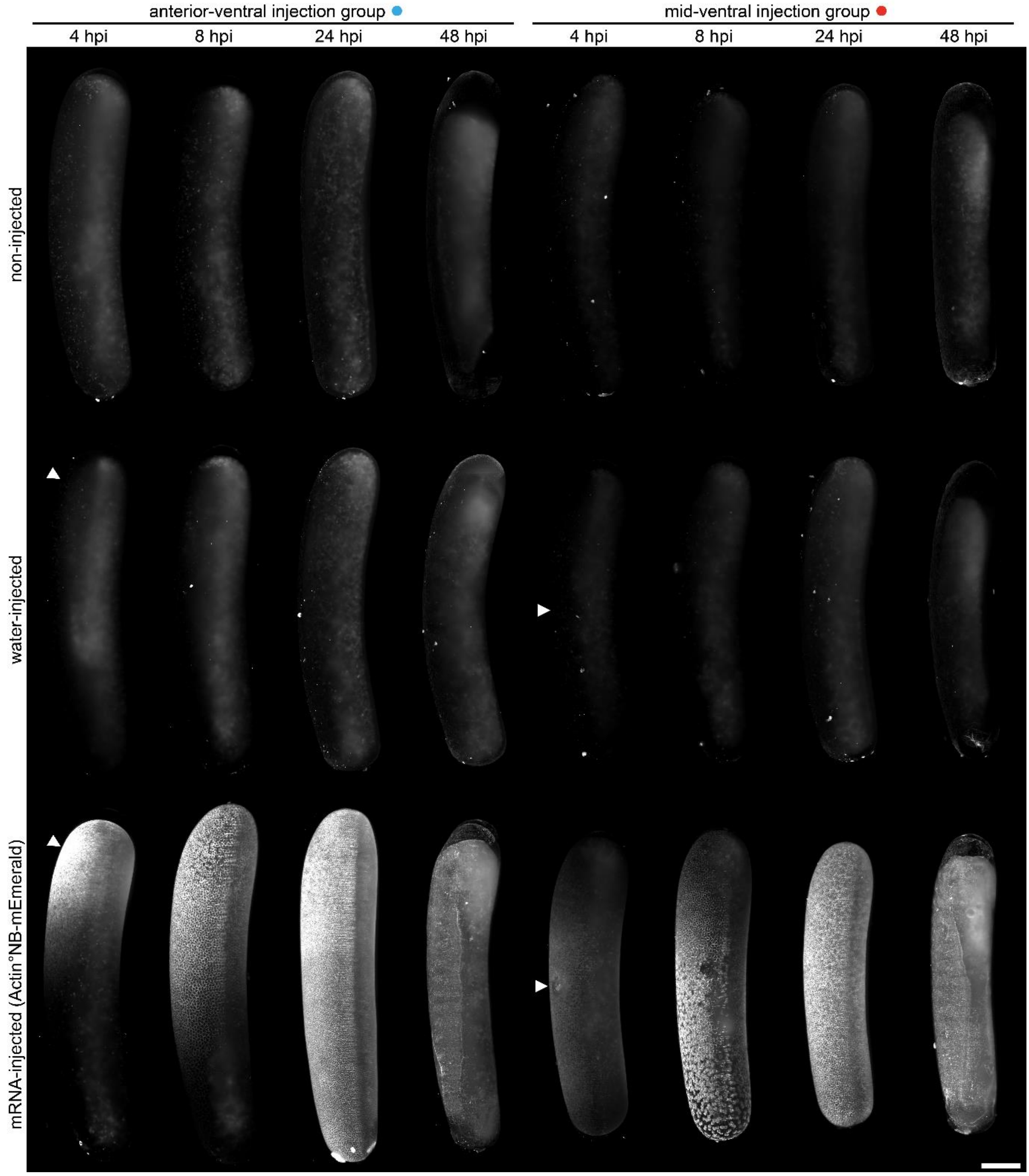

Fig. S2. Comparison of fluorescence intensity of non-injected, water-injected and mRNA-injected honeybee embryos. Distribution of fluorescence signal at different developmental stages after injection (denoted as hours post injection, hpi) at either the anterior-ventral (blue) or the mid-ventral (red) region. The non-injected and water-injected embryos show only weak autofluorescence at all investigated time points. In contrast, the embryos injected with mRNA encoding mEmerald-labeled anti-actin nanobodies show regionalized signal at 4 hpi and 8 hpi and strong uniform signal at 24 hpi and 48 hpi. Scale bar: 200 µm.

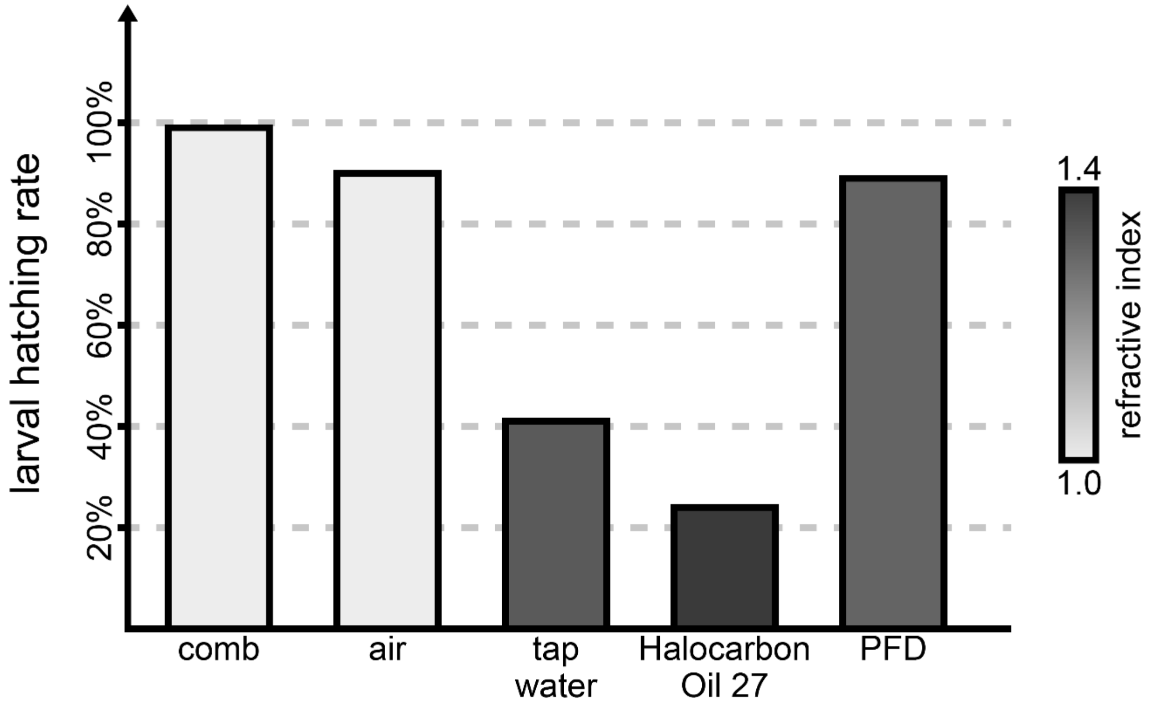

Fig. S3. Survival of honeybee embryos in various media. Honeybee embryos (between 76 and 91 individuals per condition) were incubated (i) undisturbed within their wax comb cells (close-to-natural-conditions control), or in Petri dishes in (ii) air with ≥80% relative humidity, or filled with either (iii) tap water, (iv) Halocarbon Oil 27, or (v) Perfluorodecalin (PFD) at 34.5°C. Hatched larvae were counted after 76:00 h. The data shows a general drop in survival rate for all conditions in which the embryo is removed from the comb, but also indicates that incubation in PFD achieves similar survival rates compared to incubation in air (technically closest to incubation in comb, drop in survival possibly creditable to handling, not to incubation method).

Biology Open • Supplementary information

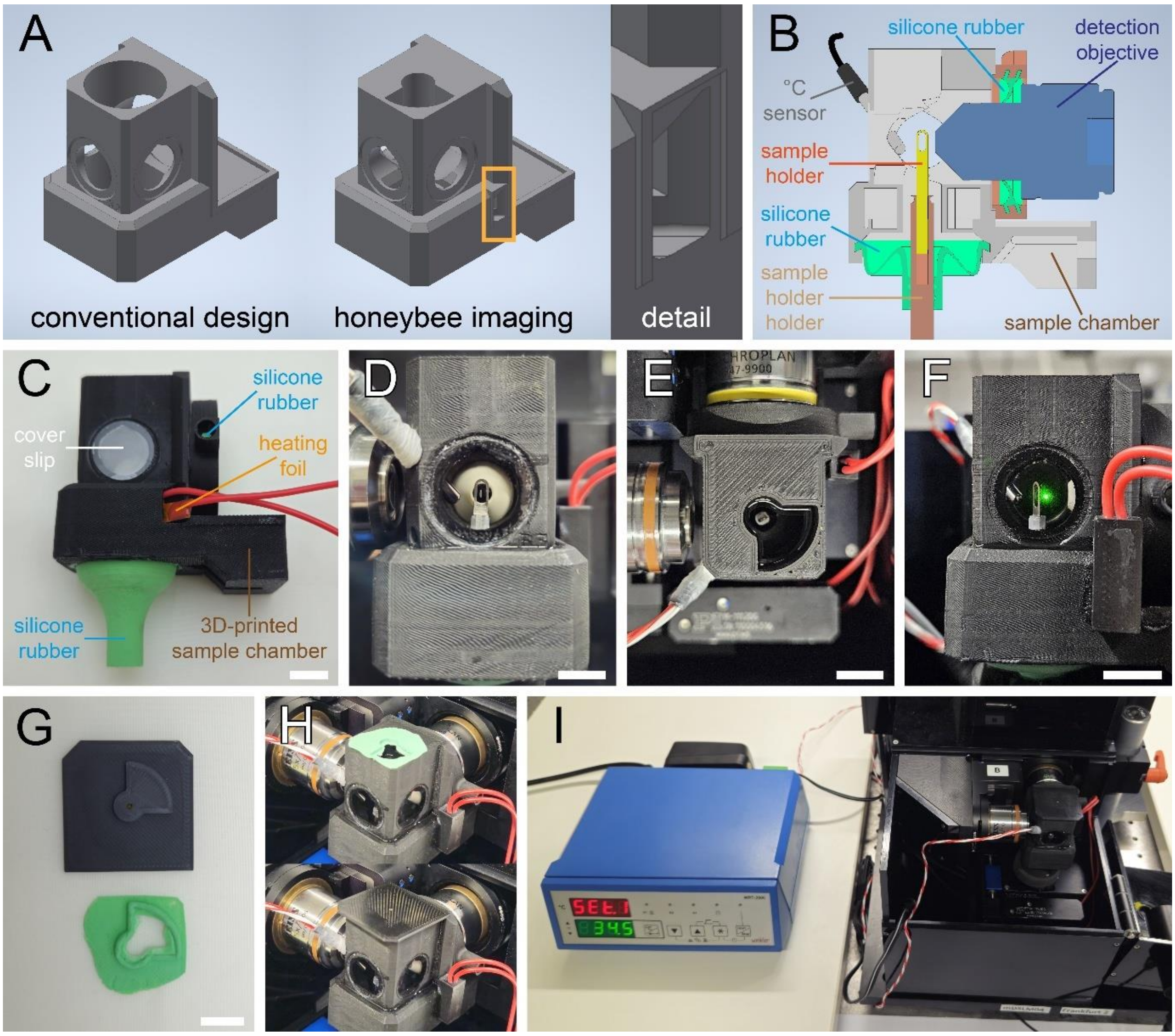

Fig. S4. Novel microscopy sample chamber design for honeybee embryo live imaging. (A) Conventional sample chamber design (example) in comparison to the customized chamber design optimized for honeybee live imaging. The volume required to fill the chamber to a convenient level has been reduced by decreasing the inner volume of the chamber. Further, a C-shaped 'heating cabinet' has been added to the base of the chamber, which allows insertion of a heating foil (side opening marked by the orange rectangle and enlarged in the detail image). **(B)** Cross-section through the customized chamber. The scheme also shows the water-dipping detection objective protruding into the sample chamber, the 'cobweb' sample holder (Strobl et al., 2017), the sample holder holder, which is attached to the microtranslation/rotation stages located below the chamber (not shown), the temperature sensor as well as two custom-made silicone rubber sealings. The hollow spaces in the base of the sample chamber indicate the heating cabinet. **(C)** 3D-printed customized sample chamber (PET). Glass coverslips are inserted into the portholes, the silicone rubber sealings are attached and the heating foil is inserted into the heating cabinet. **(D-F)** Fully assembled customized sample chamber attached to the microscope. A honeybee embryo mounted on the cobweb holder was inserted

Biology Open • Supplementary information

and moved into position for imaging. The side opening of the heating cabinet has been augmented with a 'chimney' to allow water filling. **(D)** Front view of the chamber. Visible are the temperature sensor (upper left) and the illumination objective (left). **(E)** Top view of the chamber. Visible are the illumination objective (brown ring, left) and detection objective (yellow ring, top). **(F)** Side view of the chamber. Visible are the laser (green dot in the center) and the chimney of the heating cabinet with the protruding cables (right). **(G)** Customized chamber lid (top) with silicone rubber sealing (bottom) to reduce evaporation. **(H)** Diagonal view of the sample chamber with only the sealing (top panel) and with sealing and lid (bottom panel). **(I)** Complete microscope setup for honeybee imaging with heating unit (left, set to 34.5°C) connected to the temperature sensor and heating foil. Scale bars: 10 mm (all images).

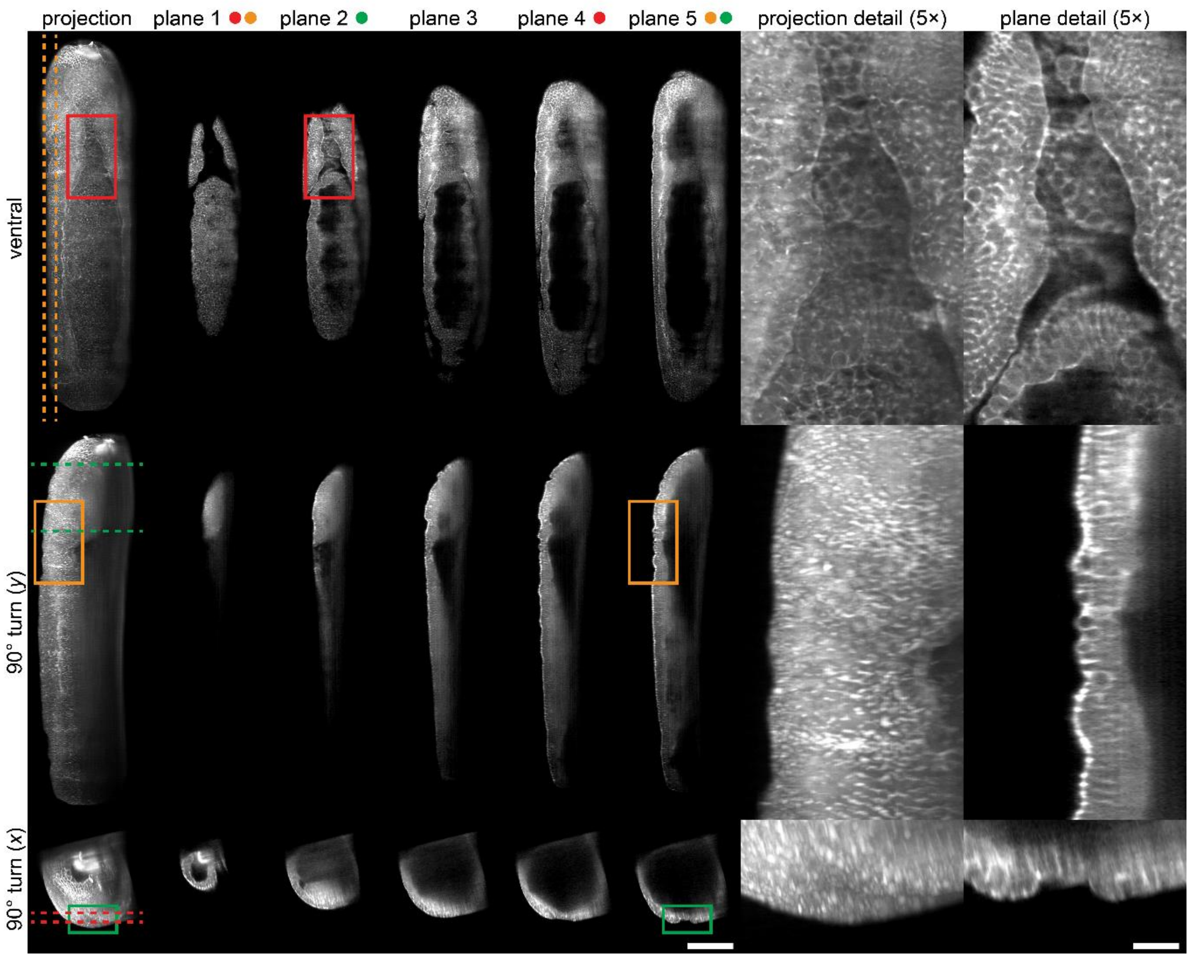

Fig. S5. Optical sections of a honeybee embryo along the ventral-dorsal axis with corresponding orthogonal sections along the lateral and anterior-posterior axes. Planes are spaced 13 µm along the ventral-dorsal and lateral axes (90° turn (*y*)) and 97 µm along the anterior-posterior axis (90° turn (*x*)). Dashed lines indicate the locations of the optical sections shown in the first, second and sixth column (marked by respectively colored dots). Detail images (positions outlined by colored rectangles) juxtapose projections and sections to illustrate the folding mesodermal tissue (first row), the ectodermal tissue sheet (second row), and midventral mesodermal furrow (third row). Scale bars: 200 µm (main images) and 40 µm (detail images).

Biology Open • Supplementary information

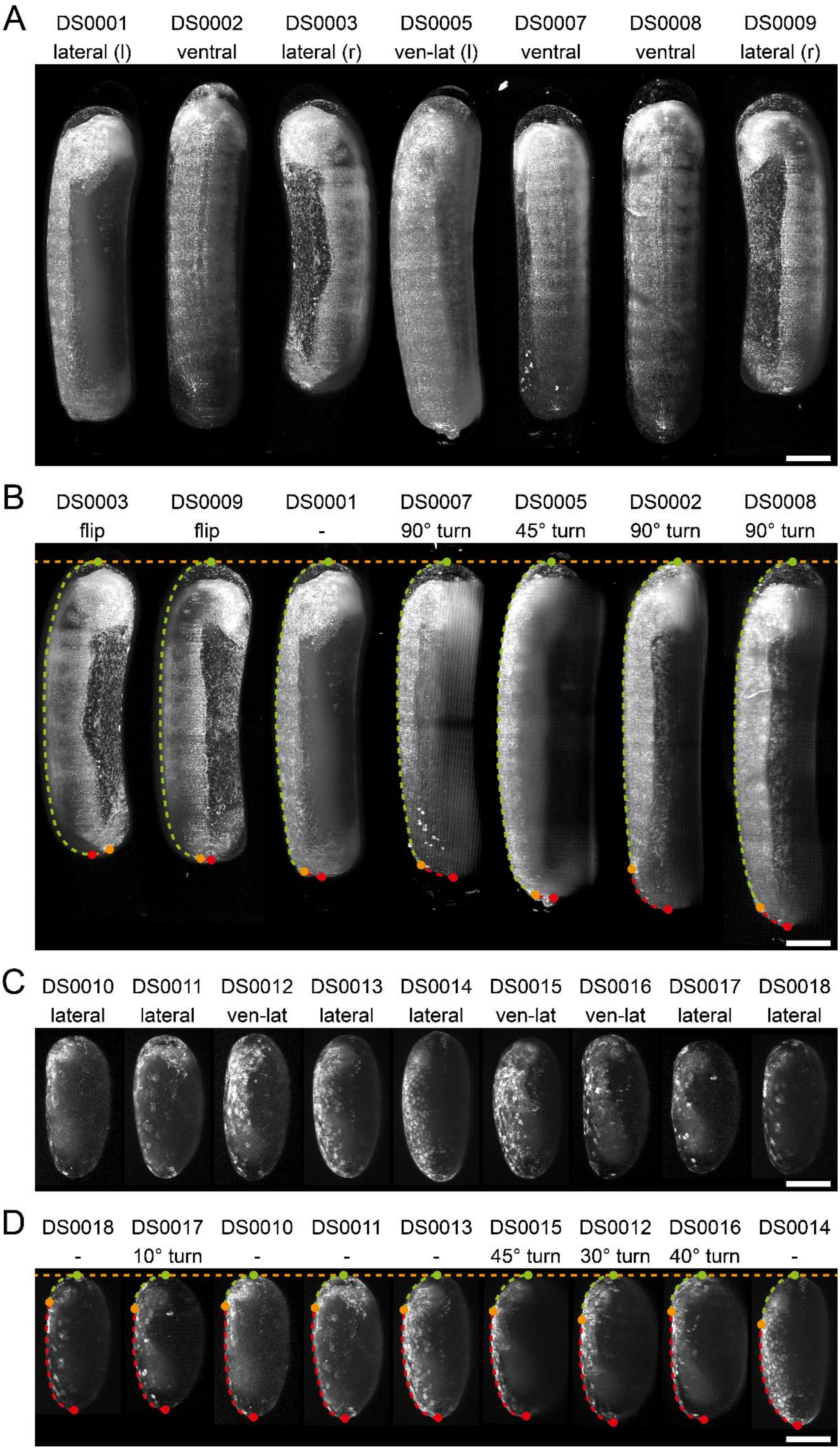
A
DS0001 lateral (l)
DS0002 ventral
DS0003 lateral (r)
DS0005 ven-lat (l)
DS0007 ventral
DS0008 ventral
DS0009 lateral (r)
B
DS0003 flip
DS0009 flip
DS0001 -
DS0007 90° turn
DS0005 45° turn
DS0002 90° turn
DS0008 90° turn
C
DS0010 lateral
DS0011 lateral
DS0012 ven-lat
DS0013 lateral
DS0014 lateral
DS0015 ven-lat
DS0016 ven-lat
DS0017 lateral
DS0018 lateral
D
DS0018 -
DS0017 10° turn
DS0010 -
DS0011 -
DS0013 -
DS0015 45° turn
DS0012 30° turn
DS0016 40° turn
DS0014 -

Fig. S6. Quantification of EEM closure locations in honeybee and red flour beetle embryos. (A) Time points of EEM window closure for seven of the nine honeybee datasets, ordered by dataset number. l, 'left' lateral view; r, 'right' lateral view. Please note that the second image of this figure (DS0002) is identical to the last image in Figure 3A (17:00 h). **(B)** Same time points after image data processing to provide lateral (l) views for all datasets, now ordered by embryo length along the ventral midline. Datasetsmarkedwith'flip'werehorizontallyflipped,datasetswith'turn'wererotatedbythe stated degrees around the *y* axis. Anterior poles of all embryos are aligned along the orange dashed line. The green dashed line indicates the distance from the anterior pole (green dot) to the EEM window closure position (orange dot) along the ventral midline, the red dashed line indicates the distance from the EEM window closure position to the posterior pole (red dot) along the ventral midline. Please note that for the first embryo, the EEM window closure point lies 'beyond' the posteriorpole. **(C)** Time points of serosa window closure for all nine red flour beetle datasets, ordered by dataset number. ven-lat, ventro-lateral view. **(D)** Same time points after image data processing to provide lateral views for all datasets, now ordered by embryo length along the ventral midline. Datasets marked with 'turn' were rotated by the stated degrees around the *y* axis. Color marks as described in (B). Scale bars: 200 µm.

Biology Open • Supplementary information

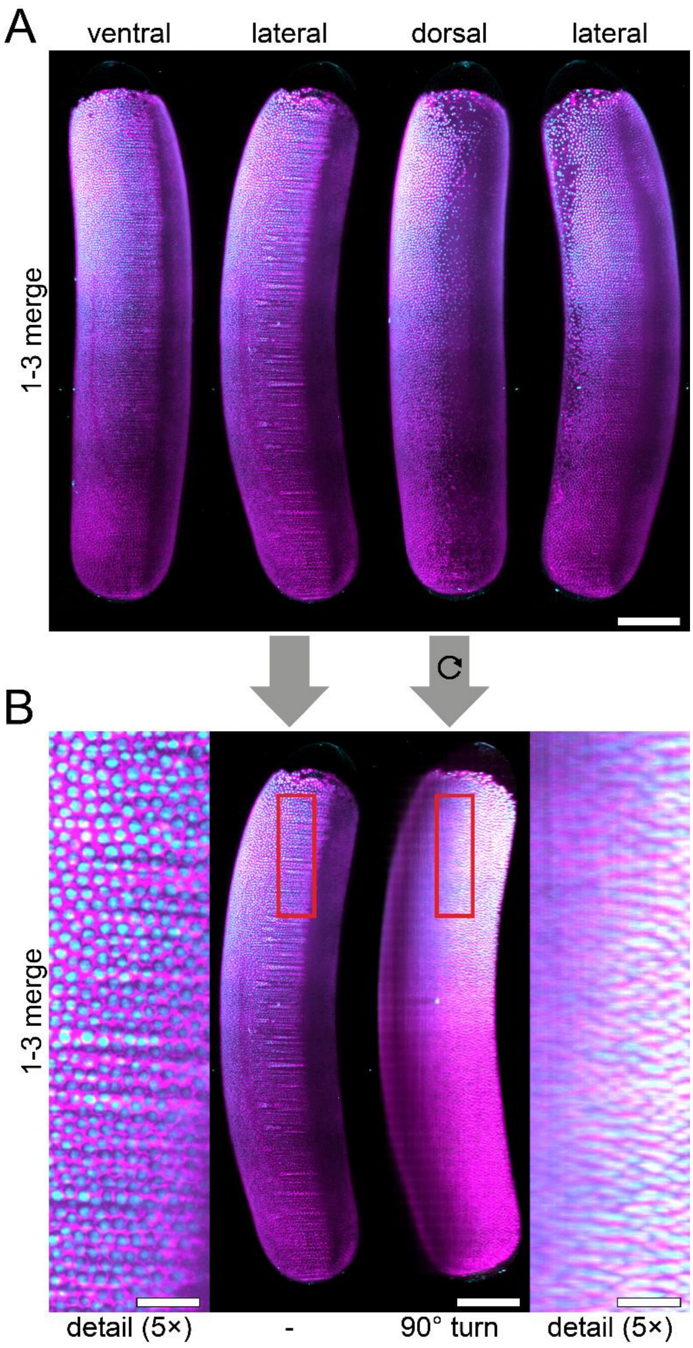

Fig. S7. Imaging of honeybee embryos along four directions. (A) The same embryo as in Figure 4 is shown here in the 1-3 merge only (mTagBFP2-labeled nuclear localization sequence tag in cyan, mCherry-labeled GAP43 membrane anchor tag with an extended linker sequence in magenta). **(B)** Juxtaposition of a *z* maximum projection from the lateral view with an orthogonal maximum projection along the *x* axis deriving from the dorsal view. Detail images (positions outlined by red rectangles) highlight anterior-lateral regions under roughly similar but ‘directionally inverted’ illumination (a direct consequence of perpendicular illumination in single-sided illumination/single-sided detection light sheet fluorescence microscopes). Scale bars: 200 µm (main images) and 40 µm (detail images).

Biology Open • Supplementary information

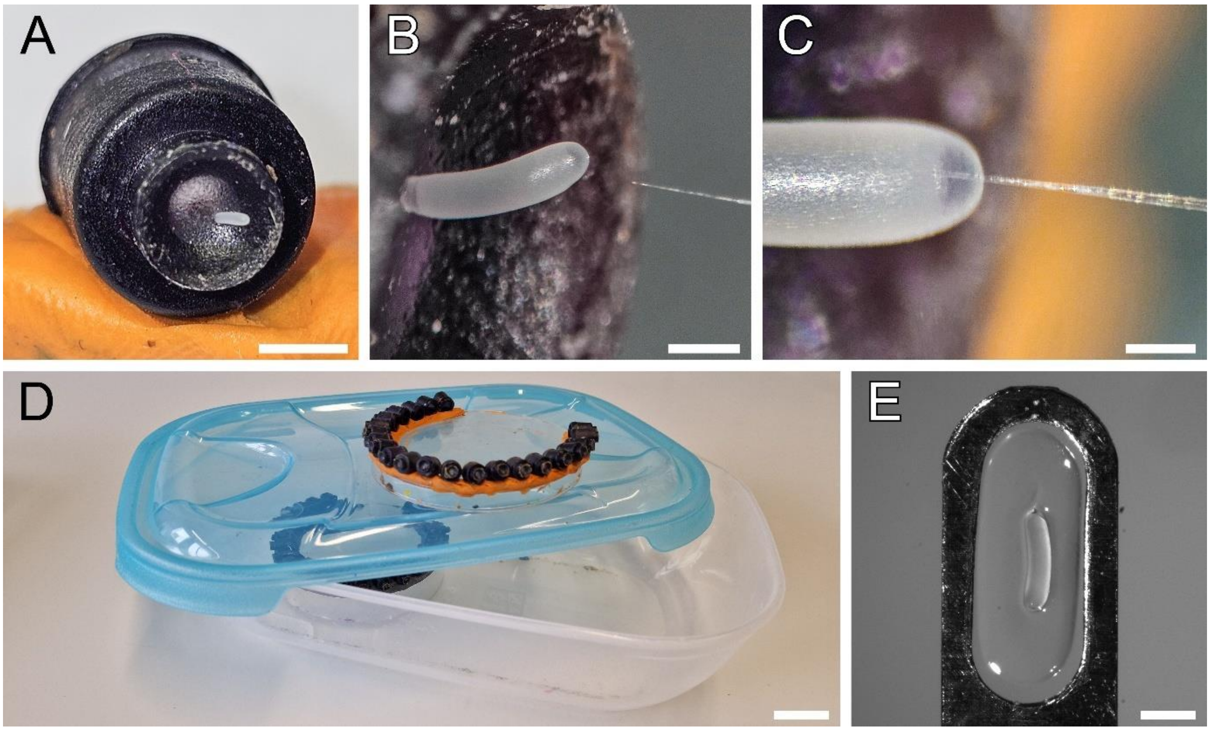

Fig. S8. Honeybee embryo injection and mounting. (A) A honeybee embryo attached to a plastic comb plug. Scale bar, 3 mm. **(B)** Lateral perspective of a honeybee embryo shortly before the injection process. Scale bar, 500 µm. **(C)** Ventral perspective close up of A honeybee embryo during injection into the anterior-ventral region. Scale bar, 200 µm. **(D)** Plastic box for incubation of injected honeybee embryos. The box accommodates two Petri dish lids, each holding 20–25 plastic comb plugs. The shown box has an inner volume of 2 liters and is filled with 0.5 ml 16% sulfuric acid solution. Scale bar, 10 mm. **(E)** Injected honeybee embryo mounted on the cobweb holder. Scale bar: 1 mm.

Biology Open • Supplementary information

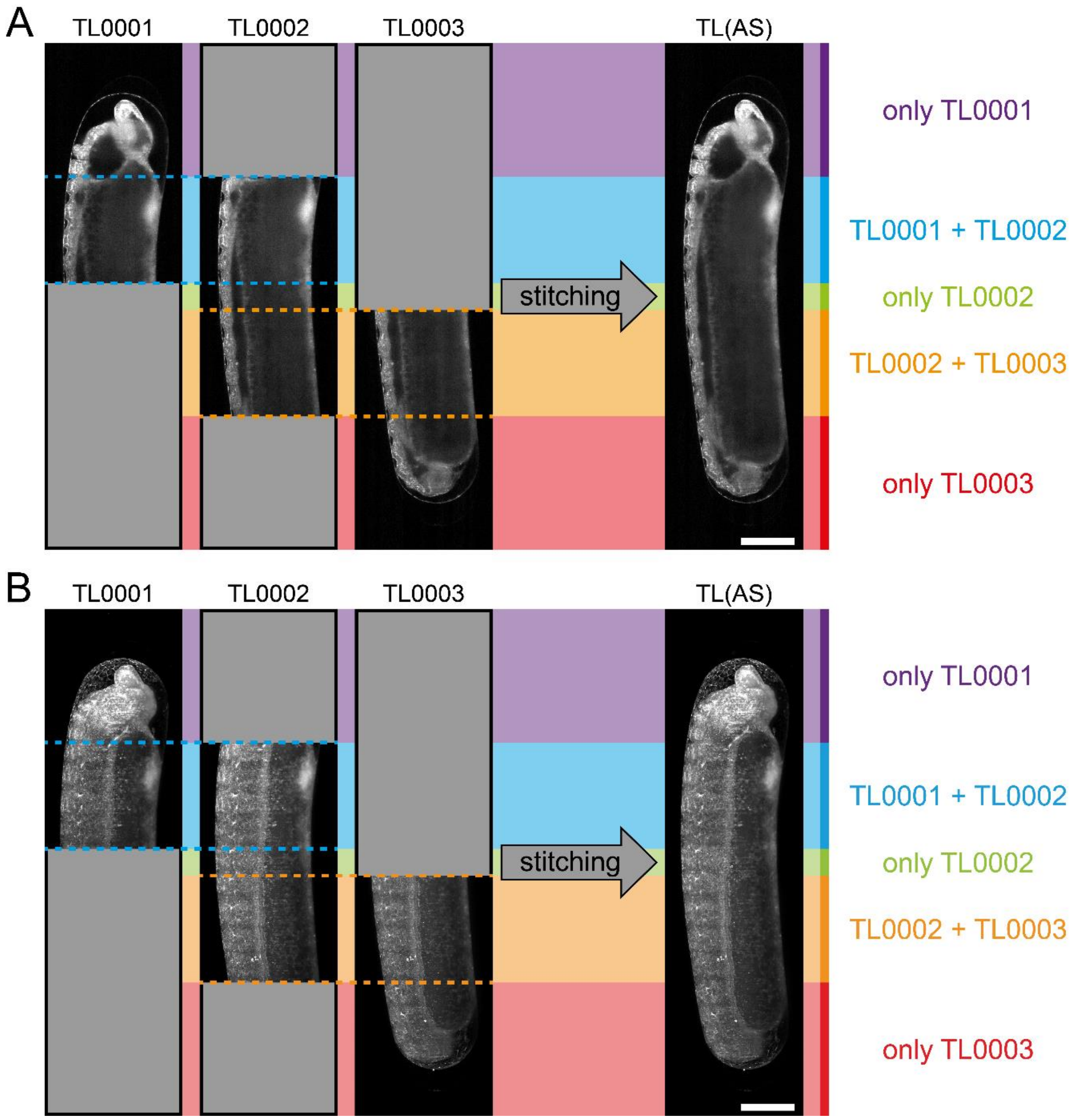

Fig. S9. Stitching of multiple tiles. (A) Using the detection objective/camera combination described in the method section, honeybee embryos were too large to be captured in one observation volume. Hence, the embryos were imaged in two to three spatially overlapping vertically arranged volumes (also known as ‘tiles’ and indicated as ‘TL’ in the file names). The tiles were ‘stitched’ into one extended volume as part of the data processing procedure using dedicated software (Preibisch et al., 2009). **(B**) Stitching of maximum projections. Scale bars: 200 µm.

Table S1. Datasets associated with this study. Several of the honeybee-associated datasets are spilt into multiple parts for convenient download.

Dataset	Species	Perspective (at the time of window closure)	Duration	Stitching	Brightness	DOI
DS0001	*Apis mellifera*	lateral (illumination ventral)	56:00 h	3 tiles	++++	10.5281/zenodo.13946167<br>10.5281/zenodo.13946187<br>10.5281/zenodo.13946233
DS0002	*Apis mellifera*	ventral	26:30 h	2 tiles	++	10.5281/zenodo.13946239
DS0003	*Apis mellifera*	lateral (illumination dorsal)	53:00 h	2 tiles	+++	10.5281/zenodo.13946250<br>10.5281/zenodo.13946266
DS0004[1]	*Apis mellifera*	dorsal	13:00 h	2 tiles	+++	10.5281/zenodo.13946276
DS0005	*Apis mellifera*	ventrolateral (illumination ventrolateral)	26:30 h	3 tiles	++	10.5281/zenodo.13946290
DS0006	*Apis mellifera*	lateral (illumination ventral)	46:30 h	2 tiles	+	10.5281/zenodo.13946304<br>10.5281/zenodo.13946311
DS0007	*Apis mellifera*	ventral	48:00 h	2 tiles	++	10.5281/zenodo.13946322<br>10.5281/zenodo.13946332
DS0008	*Apis mellifera*	ventral	37:30 h	3 tiles	++	10.5281/zenodo.13946334<br>10.5281/zenodo.13946351<br>10.5281/zenodo.13946356
DS0009	*Apis mellifera*	lateral (illumination dorsal)	49:30 h	2 tiles	++	10.5281/zenodo.13946364<br>10.5281/zenodo.13946373
DS0010	*Tribolium castaneum*	ventral, lateral, dorsal, lateral	50:00 h	-	++++	10.5281/zenodo.13950200
DS0011	*Tribolium castaneum*	ventrolateral, dorsolateral, dorsolateral, ventrolateral	37:00 h	-	++++	10.5281/zenodo.13950222
DS0012	*Tribolium castaneum*	ventrolateral, ventrolateral, dorsolateral, dorsolateral	50:00 h	-	++++	10.5281/zenodo.13950232
DS0013	*Tribolium castaneum*	ventral, lateral, dorsal, lateral	50:00 h	-	++++	10.5281/zenodo.13950242
DS0014	*Tribolium castaneum*	ventral, lateral, dorsal, lateral	37:00 h	-	++++	10.5281/zenodo.13950257
DS0015	*Tribolium castaneum*	ventrolateral, dorsolateral, dorsolateral, ventrolateral	50:00 h	-	++++	10.5281/zenodo.13950263
DS0016	*Tribolium castaneum*	ventrolateral, ventrolateral, dorsolateral, dorsolateral	50:00 h	-	++++	10.5281/zenodo.13950265
DS0017	*Tribolium castaneum*	ventrolateral, ventrolateral, dorsolateral, dorsolateral	37:00 h	-	++++	10.5281/zenodo.13950270
DS0018	*Tribolium castaneum*	ventral, lateral, dorsal, lateral	50:00 h	-	++++	10.5281/zenodo.13950275

[1]Please note that this embryo showed aberrations during development

Biology Open • Supplementary information

Table S2. Plasmids for *in-vitro* mRNA synthesis available at Addgene. All plasmids carry an ampicillin resistance cassette for amplification in dedicated *E. coli* strains. The coding sequences for the fluorescent proteins were codon-optimized for expression in the honeybee (indicated by '(AM)' in the plasmid names).

		Fluorescent protein		
		**mTagBFP2 (Subach et al., 2011) (eqFP578 derivate, excitation peak 399 nm, emission peak 454 nm)**	**mEmerald (Shaner et al., 2005) (avGFP/EGFP derivate, excitation peak 487 nm, emission peak 509 nm)**	**mCherry (Shaner et al., 2004) (DsRed derivate, excitation peak 587 nm, emission peak 610 nm)**
**Intracellular localization**	**Nucleus via the nuclear localization sequence (NLS) tag from SV40**	pBSII-NLS-mTagBFP(AM) (4,661 bp) Addgene ID: 244808	pBSII-NLS-mEmerald(AM) (4,664 bp) Addgene ID: 244811	pBSII-NLS-mCherry(AM) (4,664 bp) Addgene ID: 244814
	**Actin cytoskeleton via the Lifeact (LA) tag from yeast**	pBSII-LA-mTagBFP(AM) (4,685 bp) Addgene ID: 244809	pBSII-LA-mEmerald(AM) (4,688 bp) Addgene ID: 244812	pBSII-LA-mCherry(AM) (4,688 bp) Addgene ID: 244815
	**Membranes via the membrane anchor (MEME) tag from the human GAP43 protein**	pBSII-MEME-mTagBFP(AM) (4,730 bp) Addgene ID: 244810	pBSII-MEME-mEmerald(AM) (4,733 bp) Addgene ID: 244813	pBSII-MEME-mCherry(AM) (4,733 bp) Addgene ID: 244816

Table S3. Summary of hatching rates assayed during this study. PFD, Perfluorodecalin.

**Injection strategy**		**Condition / imaging medium**					**Injection, mounting and light sheet fluorescence microscopy-based imaging in PFD**
**Anterior-ventral**	**Mid-ventral**	**Wax comb**	**Air with ≥80% relative humidity**	**Tap water**	**Halocarbon Oil 27**	**PFD**	
63% ± 12%	40% ± 11%	99%	90%	41%	24%	89%	28%

Table S4. Metadata and imaging parameters for the long-term live imaging datasets DS0001–DS0018.

Available for download at
https://journals.biologists.com/bio/article-lookup/doi/10.1242/bio.062151#supplementary-data

Biology Open • Supplementary information

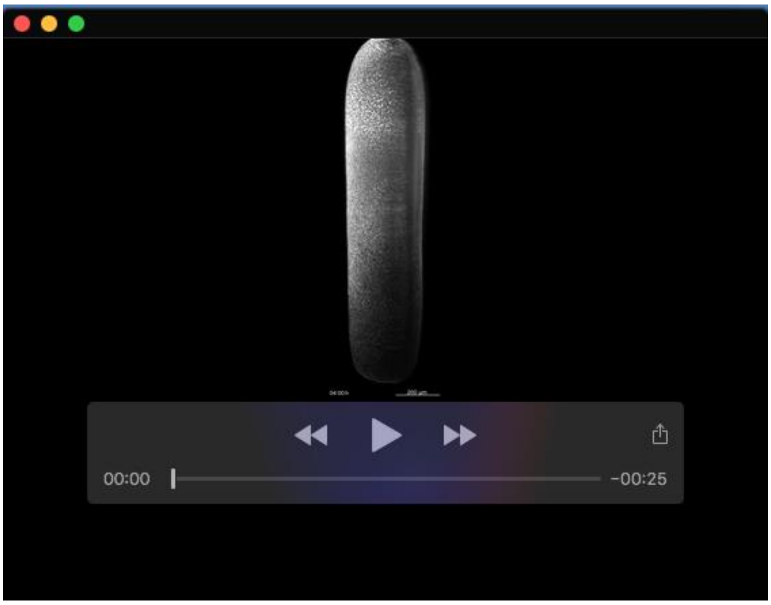

Movie 1. Long-term live imaging of a honeybee embryo injected with mRNA encoding mEmerald-labeled anti-actin nanobodies. Embryogenesis is shown along the lateral side (illumination ventral) from 00:00–56:00 h with an interval of 0:30 h between the time points. The video is based on DS0001. Frame rate is five frames per second.

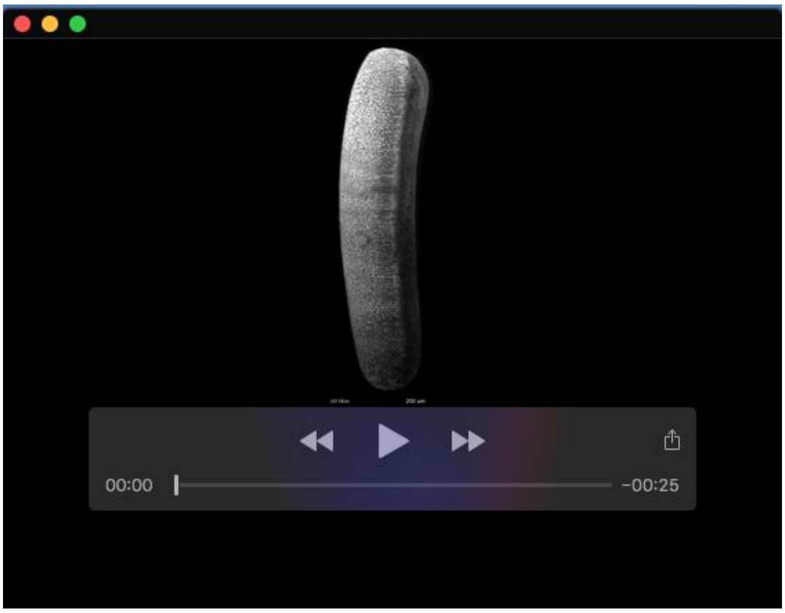

Movie 2. Long-term live imaging of a honeybee embryo injected with mRNA encoding mEmerald-labeled anti-actin nanobodies. Embryogenesis is shown along the ventral side from 00:00–26:30 h with an interval of 0:30 h between the time points. The video is based on DS0002. Frame rate is five frames per second.

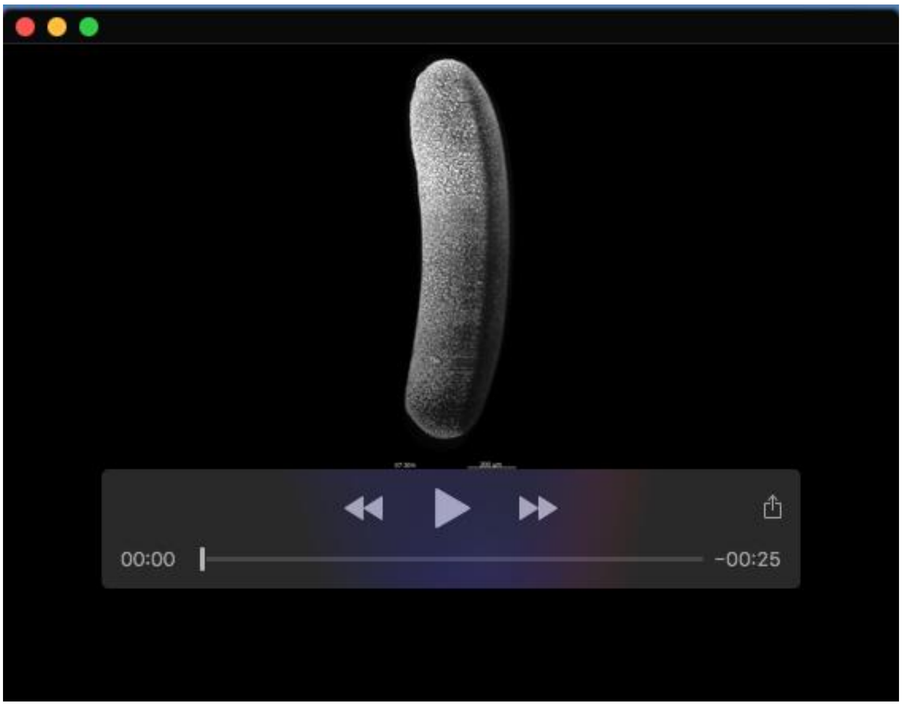

Movie 3. Long-term live imaging of a honeybee embryo injected with mRNA encoding mEmerald-labeled anti-actin nanobodies. Embryogenesis is shown along the ventral side (illumination dorsal) from 00:00–53:00 h with an interval of 0:30 h between the time points. The video is based on DS0003. Frame rate is five frames per second.
